# Nanofilament-Coated Membranes with Enhanced Scaling and Biofouling Resistance for Membrane Distillation

**DOI:** 10.1021/acsami.5c01758

**Published:** 2025-04-15

**Authors:** Mariana D. Sosa, Ivana K. Levy, Hans-Jürgen Butt, Michael Kappl

**Affiliations:** † Department of Physics at Interfaces, Max Planck Institute for Polymer Research, Ackermannweg 10, Mainz 55128, Germany; ‡ Instituto de Química Física de Materiales, Ambiente y Energía (INQUIMAE). Consejo Nacional de Investigaciones Científicas y Técnicas (CONICET)-Universidad de Buenos Aires (UBA), Ciudad Universitaria, Pabellón 2, Ciudad Autónoma de Buenos Aires C1428EGA, Argentina

**Keywords:** membrane distillation, superhydrophobicity, scaling, biofouling, silicone nanofilaments

## Abstract

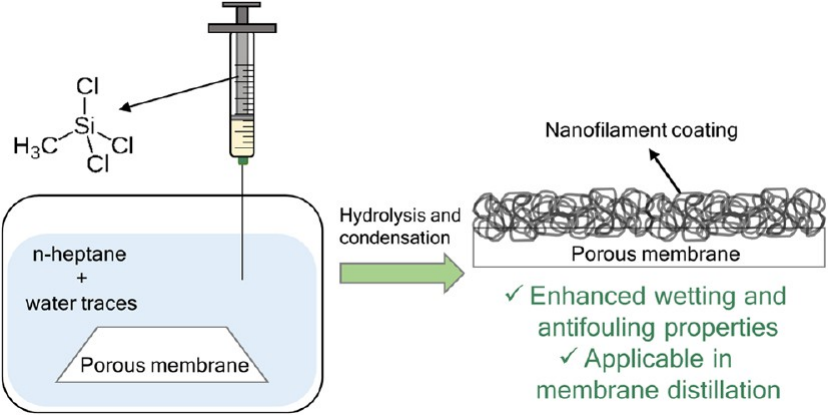

Membrane distillation (MD) for water treatment can be applied in high salinity conditions and for treatment of wastewater. Current commercial membranes are made of fluorinated polymers such as polytetrafluoroethylene (PTFE). Here, porous membranes were coated with a silicone nanofilament layer to obtain a superhydrophobic and fluorine-free material. The classical coating procedure involves the use of toluene as a solvent. In this work, *n*-heptane was tested as a less toxic alternative. Different porous membranes were tested as the substrates of the nanofilament coating. The effect of acids, scaling solutions, and biofilm formation was analyzed in comparison to standard PTFE membranes. We demonstrate that superhydrophobic nanofilament-coated poly(ether sulfone) membranes (NF-PES) possess the required antiwetting properties for MD. Moreover, NF-PES membranes have static contact angles between 10 and 20° higher than PTFE standard membranes after immersion tests in solutions containing scaling substances, and biofilm grows from 20 to 50%, less in NF-PES than in PTFE.

## Introduction

In recent years, membrane distillation has emerged as a promising and efficient technology for various separation processes for aqueous media.^1−11^ In a typical membrane distillation module, a feed solution is brought into contact with a porous membrane. The elevated temperature of the feed leads to the generation of water vapor that diffuses through the membrane and condenses on the other side. The temperature difference, which generates a vapor pressure difference across the membrane, is the driving force of the process. Typically, temperature differences of 20–40 °C are applied,^12^ so that low-grade thermal energy can be used. This process imposes requisites that the ideal membrane needs to fulfill: (i) the membrane should be wetting resistant when it is in contact with the feed solution, (ii) the porosity of the membrane has to be as large as possible for maximizing vapor permeation flux, (iii) the membrane should withstand harsh chemicals that might be present in the feed, (iv) it should ideally resist any kind of fouling (scaling, organic, or biofouling), and (v) the membrane fabrication procedure should be suitable for large-scale, environmentally friendly production.

In membrane distillation technology, two relevant difficulties lead to the necessity of developing a new generation of superhydrophobic membranes: the relatively low wetting resistance of the current membranes, which causes a decrease in clean water production, and the use of fluorine-based materials for fabricating them,^13−15^ which may no longer be acceptable in the future due to the related environmental hazards.^16,17^ Nguyen et al.^18^ explained that usual commercial membranes are made of polymers such as polypropylene (PP), polyethylene (PE), polyvinylidene fluoride (PVDF), or polytetrafluoroethylene (PTFE). While the first two exhibit low performance in MD, even at high temperatures, PVDF has a low contact angle (around 90°) and PTFE has the major disadvantage of being a fluorine-containing polymer. The authors also remark that fluorination of poly(ether sulfone) (PES), polysulfone (PSF), and ceramic membranes was also considered, but the use of fluoroalkyl agents is still necessary, and therefore, those methods are not environmentally friendly. Considering the particular case of fluoropolymers, such as PTFE, Henry et al.^19^ pointed out that these materials satisfy the criteria to be accepted as “polymers of low concern” (PLC). However, in more recent works, Lohmann et al.^20,21^ have questioned that by investigating the contribution to environmental pollution and the potential risks to humans of fluoropolymers taking into account the whole life cycle of these polymers: from the use of PFAS as processing aids in fluoropolymers production to their effects as plastic pollutants due to their persistence in the environment. As an example, the authors say that PFAS were found in concentrations around 0.3–24 ppm in personal care products that contain PTFE fine particles, which may be a relevant concentration from the toxicity point of view if compared to the maximum PFAS concentration allowed in drinking water in different countries, in the order of 10 to 100 ng/L. Moreover, they remark there is a gap of knowledge in the impact that fluoropolymers may have to the environment, which is an issue that must be considered in the field of membrane distillation as well, if clean water production is pursued.

In addition, fouling represents a major concern as in many other membrane technologies. Traditionally, biofouling has been neglected in membrane distillation because bacteria are not expected to grow in a medium with high salinity and temperature. However, several authors pointed out that microorganisms that survive under extreme conditions may develop biofilms, which decreases the vapor flux across the membrane.^22−28^ In addition, increasing attention is being paid to scaling. Carbonate and sulfate crystallization may contribute to flux decline given that their solubility decreases at high temperature.^29−31^ In a previous work from our group, the water repellency of NF-PES in contact with low surface tension liquids was quantitatively and qualitatively studied and compared to commercial PTFE membranes. That provided valuable information about the range of applicability of the membranes and it has been very well established that NF-PES is more resistant to organic fouling than current commercial alternatives for membrane distillation.^32^ In Madalosso et al.,^33^ an extensive investigation of the state-of-the-art membrane modification procedures was conducted. Plasma treatment, electrospinning, and coating are identified as the most common methods for modifying commercial membranes in order to enhance the vapor permeation flux and the liquid entry pressure (LEP). Interestingly, the majority (around 80%) of modified membranes in the current scientific scenario only explore classical desalination processes (NaCl solutions) and organic foulants, such as oils, surfactants, and other low surface tension liquids. In this case, evaluating the resistance toward acids is relevant for applications in industrial wastewater treatment, while the effect of scaling solutions is relevant for most of applications, given that scaling is a very ubiquitous yet nondesirable effect in most of membrane technologies.

Silicone nanofilament-coated membranes appear attractive due to their excellent water repellency.^34−37^ It has been proven that silicone nanofilament-coated membranes are promising superhydrophobic materials with enhanced clean water production in comparison to polytetrafluoroethylene (PTFE) and polyethylene (PE) commercial membranes.^38^ Moreover, the nanofilament-coated membranes are fluorine free, being more environmentally friendly than usual membranes for membrane distillation.

The traditional nanofilament coating process involves activation of the surface for generating −OH groups that are available for further modification. The activation is typically conducted by an oxygen plasma treatment. Once the reactive groups are generated, in a second step, silicone nanofilaments are grown from trichloromethylsilane (TCMS) dissolved in toluene. The toluene contains trace amounts of water. TCMS has three chlorine atoms that may be hydrolyzed by traces of water present in the organic solvent to give rise to the corresponding silanol. Then, condensation of silanol and −OH surface groups occurs, which results in a first layer of siloxanol. The consequent polymerization of TCMS from the siloxanols on the surface finally leads to the formation of a nanofilament-like structure.^36,39^

Choosing the appropriate solvent for nanofilament synthesis is essential.^40^ For example, when highly polar solvents are used, the water content is large, and therefore TCMS polymerizes as an extended flat coating or as a particle-like coating. The situation changes when low polarity solvents are used with low water content (up to 200 ppm). In this case, TCMS polymerizes with a nanofilament-like shape. It was proposed that the traces of water in the solvent form nanodroplets on top of the original substrate. Hydrolysis and condensation of TCMS occur within the droplets, which assist the nanofilaments growth.^37^ It means the solvent itself and its water content are key parameters when preparing silicone nanofilaments.^6^ Typically, the coating reaction is carried out using toluene with a water content of around 190 ppm.

For industrial applications, nonaromatic solvents are preferred. It implies that the upscaling is, to some extent, confronted with the optimum conditions for growing the nanofilament structure. In this work, nanofilament-coated membranes were prepared using *n*-heptane saturated with water (90 ppm) instead of aromatic solvents. Concerning the toxicity of organic solvents used for nanofilaments growth, as far as we know there are no specific studies comparing the toxic effects of toluene and *n*-heptane, but it is generally accepted that aromatic substances are more hazardous than aliphatic ones. Also, the minimum narcotic concentration and fatal concentration for *n*-heptane are higher than toluene.^41^ Toluene is known to cause persistent effects in the central nervous system while no neurological effects were found in studies done with cyclohexane, *n*-heptane, and branched isomers of hexane. Among aliphatic compounds, *n*-hexane seems to be significantly more toxic than other members of C5–C10 family compounds, since a highly dangerous metabolite is created during its metabolism (2,5-hexanedione).^42^ In summary, *n*-heptane seems to be a reasonable alternative to toluene and other aromatic solvents that may be suitable for nanofilament synthesis at a lab scale but not in large production.

Poly(ether sulfone) porous membranes of two different pore sizes were used as substrates, and the wetting properties of the coated membranes were studied.

Two relevant applications of membrane distillation are desalination and ammonia recovery from aqueous digestates.^3,12,43−47^ Therefore, membrane distillation performance was explored in two different cases: using synthetic seawater containing scaling substances and a real digestate water sample containing ammonia.

In a previous work, we reported that silicone nanofilament-coated membranes show liquid repellency in contact to low surface tension solutions and are more resistant to organic fouling in comparison to commercial membranes.^32^ This work aims to provide an alternative to the currently used membranes described above, considering the following advantages: (i) nanofilament-coated membranes are fluorine free; (ii) a nonaromatic solvent can be used; (iii) higher resistance to inorganic and biofouling was achieved; and (iv) the coating procedure has potential for upscaling due to its simplicity.

## Materials and Methods

### Chemicals and Membranes

Trichloromethylsilane (TCMS) and *n*-heptane were supplied by Sigma-Aldrich and Honeywell, respectively. Polyether sulfone (PES) membranes (pore sizes 1.2 and 3 μm) and polypropylene (PP) membranes (pore sizes 1 and 3 μm) were supplied by Dorsan Filtration. PTFE membranes (pore size 0.2 μm) were obtained from Donaldson Filtration Solutions. Cellulose acetate membranes (pore size of 1.2 μm) were from Sterlitech Corporation.

Heptane was saturated with water by adding approximately 10 mL of distilled water to 1 L of heptane and stirring both phases at 800 rpm overnight in a bottle. Afterward, the stirring was interrupted, and the phases spontaneously separated. The final water content in heptane was 90 ppm, determined by Karl Fischer titration. For simplicity, water-saturated *n*-heptane will be referred to just as heptane from now on. The rest of the reagents and materials were used as received.

### Fabrication of Silicone Nanofilament Coatings

**Figure 1:**
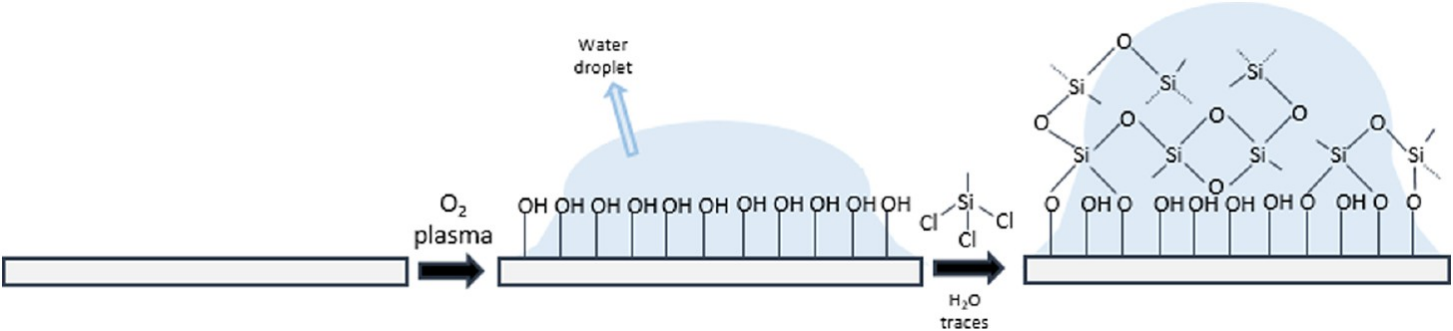
Scheme of the mechanism of silicone nanofilament growth. Plasma treatment generates active surface groups increasing surface hydrophilicity allowing the formation of water nanodroplets. Within the droplets, hydrolysis and condensation of TCMS occur guided by the droplets, conducting to a final nanofilament-like structure.

The coating process was performed in TCMS:heptane 17 mM solution. To generate −OH groups on the surface, oxygen plasma activation of the original membranes’ surface was carried out in a Diener Electronic Femto plasma device at 0.6 mbar oxygen pressure and 90 W for 2 min. After the plasma treatment, surface active −OH groups were obtained. Then, the membranes (7.2 × 6 cm^2^) were immersed in 250 mL of the TCMS:heptane solution. The coating reaction was conducted overnight. Finally, the samples were rinsed with pure hexane and dried at room temperature. Figure 1 shows how the coating reaction proceeds. The first step (plasma treatment) generates active surface groups. Once the membrane is immersed in heptane-containing traces of water, small droplets form on the membrane’s surface. It is inside the droplets that hydrolysis and condensation of TCMS occur. Therefore, polymerization of TCMS is guided by the water droplets, and they determine the nanofilament shape of the coating.

### Characterization of the Membranes

Static contact angles of the membranes were measured with a DataPhysics OCA35 goniometer in ‘sessile drop’ mode with 6 μL water drops on five different spots of each surface. The contact angle was obtained by an elliptic fitting of contour images of the drop, and the contact angle on the right and the left was recorded. Contact angle hysteresis was determined using the same equipment in ‘sessile drop with needle in’ mode. Advancing and receding contact angles were measured twice in five different spots on the membranes’ surface. For measuring advancing contact angle, an increase in the volume of the drop from 6 to 36 μL at a dosing rate of 0.5 μL/s was used. For the receding contact angle, the volume of the drop was decreased back to 6 μL at the same rate.

Liquid entry pressure of the membranes was measured with a syringe pump connected to a pressure transducer and a tailored membrane holder. A 50 mL Hamilton syringe was filled with water and placed in a syringe pump. Then, the liquid was pumped through the pressure transducer, which was connected to the membrane holder. The liquid pressure on top of the membrane was measured over time. A rapid increase in the pressure is typically observed at the beginning of the measurement, until reaching a maximum value. At this point, water leakage is observed, which means that the liquid entry pressure value has been reached.

Surface morphology was studied by scanning electron microscopy using Zeiss LEO1530 Gemini equipment. To avoid charging, the samples were coated with a 7 nm Pt layer by sputtering (CCU-010HV high-vacuum compact coating unit, Safematic GmbH).

### Membrane Distillation Experiments

Membrane distillation experiments were carried out in a laboratory scale air gap membrane distillation (AGMD) module. Distilled water was used as the coolant fluid. The coolant temperature was set at 15 °C. Different feed temperatures were tested: 35, 45, 55, 65, and 75 °C, which gives a temperature difference between the feed and the coolant (Δ*T*) of 20, 30, 40, 50, and 60 °C, respectively. Temperatures in the feed, temperature in the coolant, distillate mass, and conductivity in the distillate were continuously monitored. From these measurements, distillation flux (eq 1) and salt rejection percentage (eq 2) were calculated according to
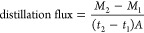
1

*M*_2_ and *M*_1_ are the distillate masses measured at times of *t*_2_ and *t*_1_. *A* is the active membrane surface (19.22 cm^2^).
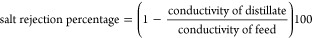
2

Salt rejection percentage is a measure of the quality of the distillate in desalination. Therefore, eq 2 was used for quantifying the quality of the distillate when using solutions containing salts.

The nanofilament-coated membranes were tested in two cases: (i) a solution with the following composition: NaCl 25 g/L, Na_2_SO_4_ 4 g/L, CaCl_2_.2H_2_O 2 g/L, and Na_2_CO_3_ 0.01 g/L (called synthetic seawater along the text) and (ii) for ammonia recovery. In that case, a real wastewater sample provided by SolarSpring GmbH was used. The feed is a digestate with ammonia concentration between 800 and 1200 mg/L, which is produced as a result of the treatment of nitrogen containing organic matter. The liquid may also contain phosphorus, potassium, and organic matter, although no quantitative data of the exact chemical composition of the sample are available. The conductivity of the solution is 500 mS/m. In this case, the recovery percentage was roughly estimated by
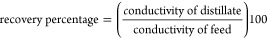
3

The following conditions were constant in all membrane distillation experiments:

- Feed flow rate: 1 L/min
- Coolant flow rate: 2 L/min
- Air gap width: 3.5 mm
- Effective membrane area: 19.22 cm^2^

### Acid Resistance and Scaling Tests

Resistance against acid media was studied by immersing the membranes in an HCl solution at pH 1 for 24 h. Wetting properties of the immersed samples were measured and compared with the nonimmersed membranes. Scanning electron microscopy images of the immersed membranes were also recorded to investigate possible modifications in the surface morphology after the immersion experiments.

For studying the effect of scaling, a synthetic seawater solution utilized in MD experiments was used. The first test involved the immersion of six coated and six noncoated membranes (prepared independently) in the synthetic seawater for 24 h at room temperature. Then, contact angle and contact angle hysteresis were measured and compared to nonimmersed membranes. The same experiment was performed for PTFE standard membranes for comparison. Scanning electron microscopy images of the immersed membranes were taken for identifying possible crystal formation on the surface.

Air gap membrane distillation (AGMD) experiments on PTFE and NF-PES membranes were conducted using synthetic seawater as the feed following the procedure described in the previous section. In addition to it, AGMD at constant Δ*T* = 60 °C for 24 h was carried out using NF-PES 3 membranes to test possible flux decline over time due to scaling.

### Biofouling Assays

Biofilm growth studies were performed with the Gram-negative bacteria *Pseudomonas protegens Pf-5*, abbreviated as Pp. The culture medium used was Luria–Bertani (LB) composed of 0.5% yeast extract and 1% tryptone (both from Britania Laboratories) and 1% sodium chloride (from Biopack), %w/v, in water.

Colonies of Pp grown on an LB agar solid plate were collected using a sterile tip and subsequently inoculated into a sterile LB liquid medium. This suspension was incubated overnight under orbital shaking (250 rpm, room temperature) to obtain a liquid culture of Pp. The OD (optical density) in the overnight *Pf-5* liquid culture was measured at 600 nm and then diluted to an OD of 0.1 in fresh LB. Estimations of bacterial viability, by the conventional method of serial dilutions and plate count, showed that OD 0.1 corresponds to CFU (colony forming units) 10^7^–10^8^.

For biofilm assays on the membranes, the coated and pristine membranes were cut in 1 cm by 1 cm pieces, sterilized by UV light for 3 min (59S UV Sanitizer box, λ = 260–280 nm, 8W), and then placed in a 24 well polystyrene plate. After these steps, 1000 μL of the suspension of OD 0.1 was placed as inoculum in each well and incubated overnight at room temperature. LB (1000 ul) without Pp was used as the negative control.

After 24 h, the percentage of biofilm growth was calculated by a modification of the classical crystal violet method for microplates adapted for surface measurements.^48^ For each sample (coated or pristine membrane), the absorbance at 600 nm was measured in a microplate reader. After subtraction of the corresponding blank for each sample (membrane with culture media without inoculum), the absorbance of the corresponding sample was determined. All of the experiments were performed at least twice on different days, using between 2 and 3 replicates of each sample, and the results were averaged. Error bars were calculated as the standard deviation of the average.

Additionally, a set of membranes before and after incubation in the same conditions (Pp OD = 0.1, *t* = 24 h, RT) were analyzed by SEM-EDS and optical microscopy. The samples were taken from the incubation plate after 24 h of incubation, rinsed with abundant water, dried with nitrogen, and stored at 4 °C until analysis.

## Results and Discussion

**Figure 2:**
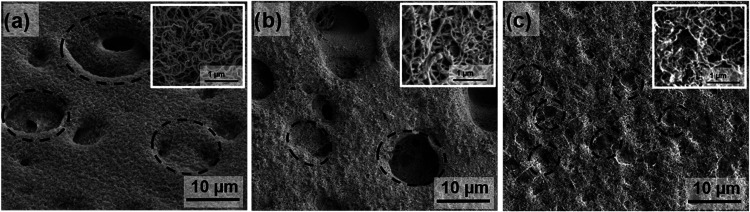
SEM images of the coated membranes. (a) Coated poly(ether sulfone) substrate (pore size: 8 μm) using toluene with 180 ppm of water as a solvent, (b) coated poly(ether sulfone) membrane (pore size: 3 μm) using *n*-heptane with 90 ppm of water as a solvent, and (c) coated poly(ether sulfone) membrane (pore size: 1.2 μm) using *n*-heptane with 90 ppm of water as a solvent. The circles indicate where the pores of the original membranes are located, and the insets are higher magnification images of the coatings.

With the aim of replacing toluene, *n*-heptane, *n*-hexane, acetone, and petroleum ether were tested, but reproducible results were only obtained when using *n*-heptane saturated with water (90 ppm). Figure 2 shows the SEM images of poly(ether sulfone)-coated membranes (NF-PES) using toluene with 180 ppm of water (Figure 2a) and *n*-heptane saturated with water (Figure 2b,c). When using toluene, silicone nanofilaments grow on top of the membrane as well as inside its porous structure. This allows the use of large pore size membranes, which enhances the vapor permeation flux, maximizing the water repellency given by the nanofilament coating.^38^ In the case of *n*-heptane, the coating appears as a dense layer on top of the surface but does not completely span all of the pores of the membrane. However, this changes significantly when decreasing the pore size down to 1.2 μm (Figure 2c) where almost full coverage of the surface is achieved and the pores of the original membrane are more difficult to spot. Variations in the coating structure may have consequences in both wetting properties and membrane distillation performance. In the following sections, experimental evidence proving that the membranes prepared using *n*-heptane fulfill the requirements to be applied in membrane distillation will be shown.

**Figure 3:**
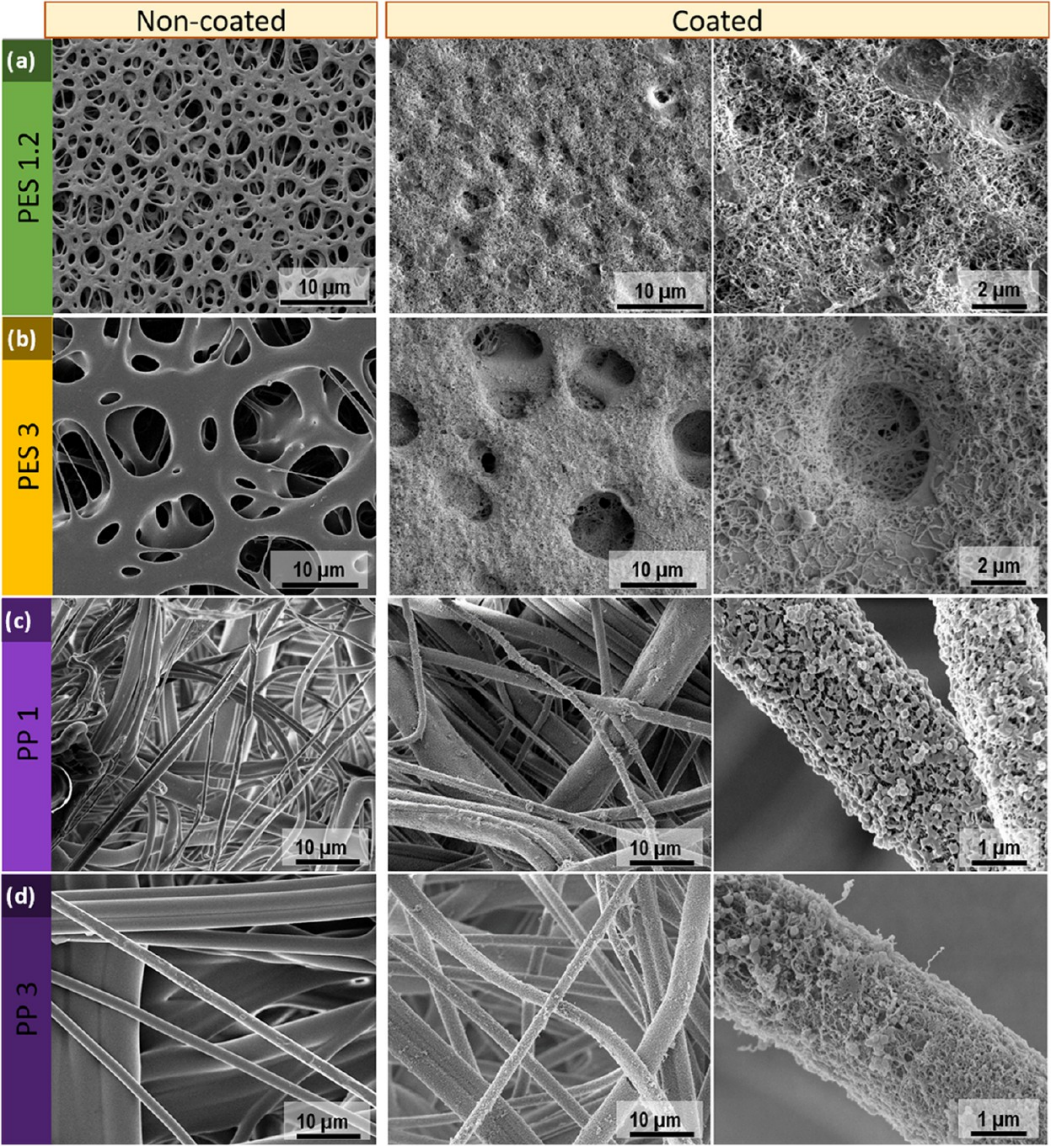
SEM images of the original (left) and coated substrates (right). Two different magnifications images of the coated samples are provided for better understanding. (a) Poly(ether sulfone) 1.2 μm of pore size, (b) poly(ether sulfone) 3 μm of pore size, (c) polypropylene 1 μm of pore size, and (d) polypropylene 3 μm of pore size. For PES membranes, the nanofilament coating grows on top of the surface and inside the pores. For PP membranes, the single PP micrometric fibers are coated but the shape is a combination between particles and filaments.

Variations in the surface morphology and hydrophobicity of the core membrane might influence the properties of the coating. For instance, when using nonwoven polypropylene membranes (PP), instead of poly(ether sulfone) (PES), the nanofilaments only grow on some spots of the fibers and are not able to form a dense layer covering the outer surface of the nonwoven substrate (Figure 3). This might be caused by the combination of the more open morphology of the surface and the more hydrophobic nature of the single PP fibers in comparison to PES membranes: if we assume the drop-guided coating mechanism proposed by the group of Seeger^37^ is valid, then the water dissolved in the solvent should form droplets on top of the membrane acting as nanoreactors for hydrolysis and condensation of TCMS. The reaction should proceed assisted by the droplets. In this context, it is reasonable that a more hydrophobic substrate such as PP hinders formation of water droplets on the surface in contrast to the more hydrophilic PES, and therefore, TCMS may react with trace water more in the solvent bulk than at the surface. In fact, plasma-treated polypropylene membranes have a static contact angle of 124° and a contact angle hysteresis of 60°, which means that despite the plasma treatment, the membranes are still hydrophobic. The opposite is expected in working with a more hydrophilic material. When cellulose acetate membranes, which are intrinsically hydrophilic and possess – OH active groups in the polymer structure, a nanofilament coating is obtained. When increasing the hydrophilicity by applying oxygen plasma, the outcome is a flat coating covering the rough structure of the cellulose acetate pristine membrane (Figure S1).

We conclude that the core membrane should be hydrophilic to some extent for ensuring the nucleation of enough water drops on the surface. In the case of hydrophilic polymers that do not contain −OH groups in the polymer structure, plasma activation prior to hydrolysis and condensation of TCMS is required. However, an excess of plasma activation could increase the hydrophilicity to the point that a flat coating is obtained, most likely due to the formation of a continuous water layer.

**Figure 4:**
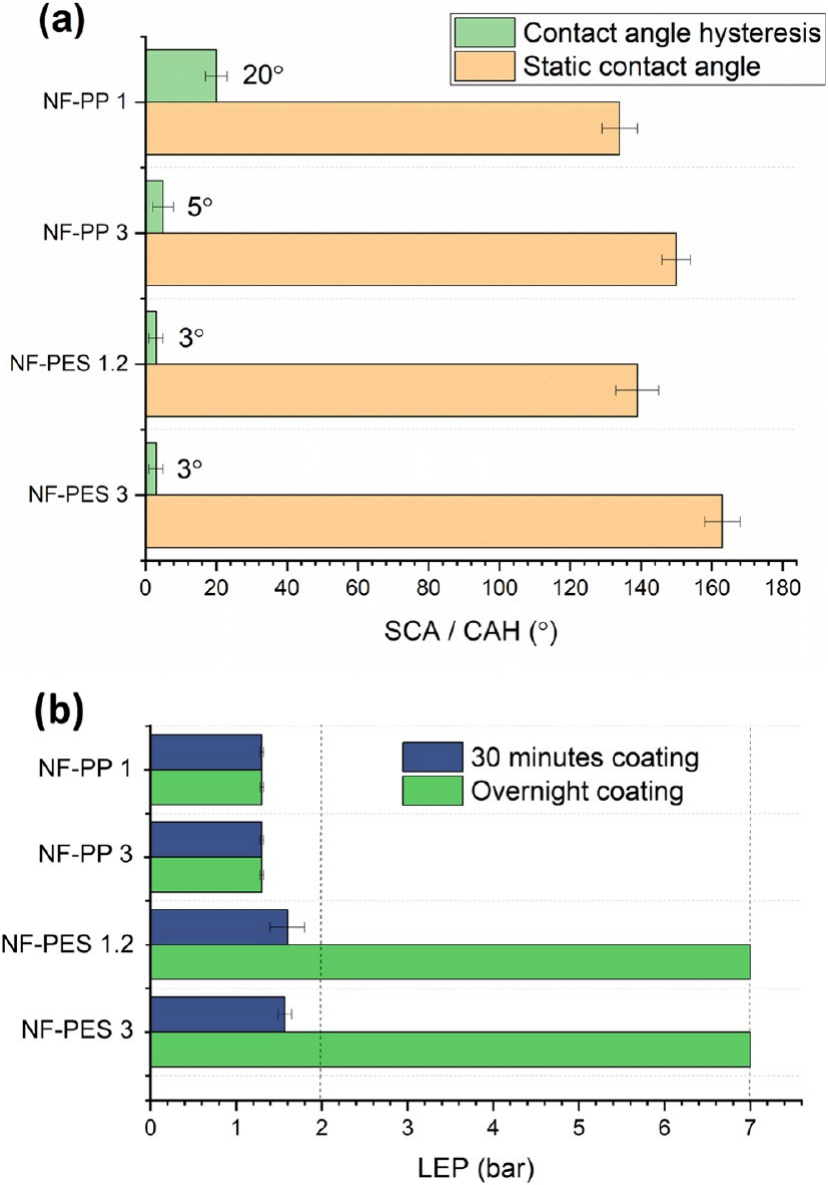
(a) Static contact angle and contact angle hysteresis of coated PES and PP membranes. (b) Liquid entry pressure of coated PES and PP membranes after conducting the coating reaction for 30 min and overnight.

Static contact angles and contact angle hysteresis values measured for both PES and PP coated membranes show that they are highly hydrophobic or superhydrophobic (Figure 4a). For the case of PP membranes, the wetting properties do not vary significantly in comparison to the noncoated samples (SCA ≈ 135° CAH ≈ 20°). In the case of PES, the changes with respect to the original membranes are dramatic since the pristine material is superhydrophilic and becomes superhydrophobic after the coating reaction.

The liquid entry pressure of nanofilament-coated PES membranes was better than the liquid entry pressure of nanofilament-coated PP membranes (Figure 4b). Even after 30 min coating, a LEP value higher than atmospheric pressure was measured. When conducting the coating overnight, a value around four times higher than on PP membranes was measured, meaning that the coating growth is fully developed. In the case of PP samples coated for 30 min and overnight, no significant differences were observed. This means that the LEP is not dependent on the coating time for PP membranes. The reaction seems to occur more in the bulk solvent than at the surface. Therefore, increasing the reaction time does not result in any improvement of the coating.

Since coated PES membranes exhibit better wetting resistance than coated PP samples, NF-PES membranes were selected for application in membrane distillation.

### Effect of Acid Solutions and Scaling Substances

**Figure 5:**
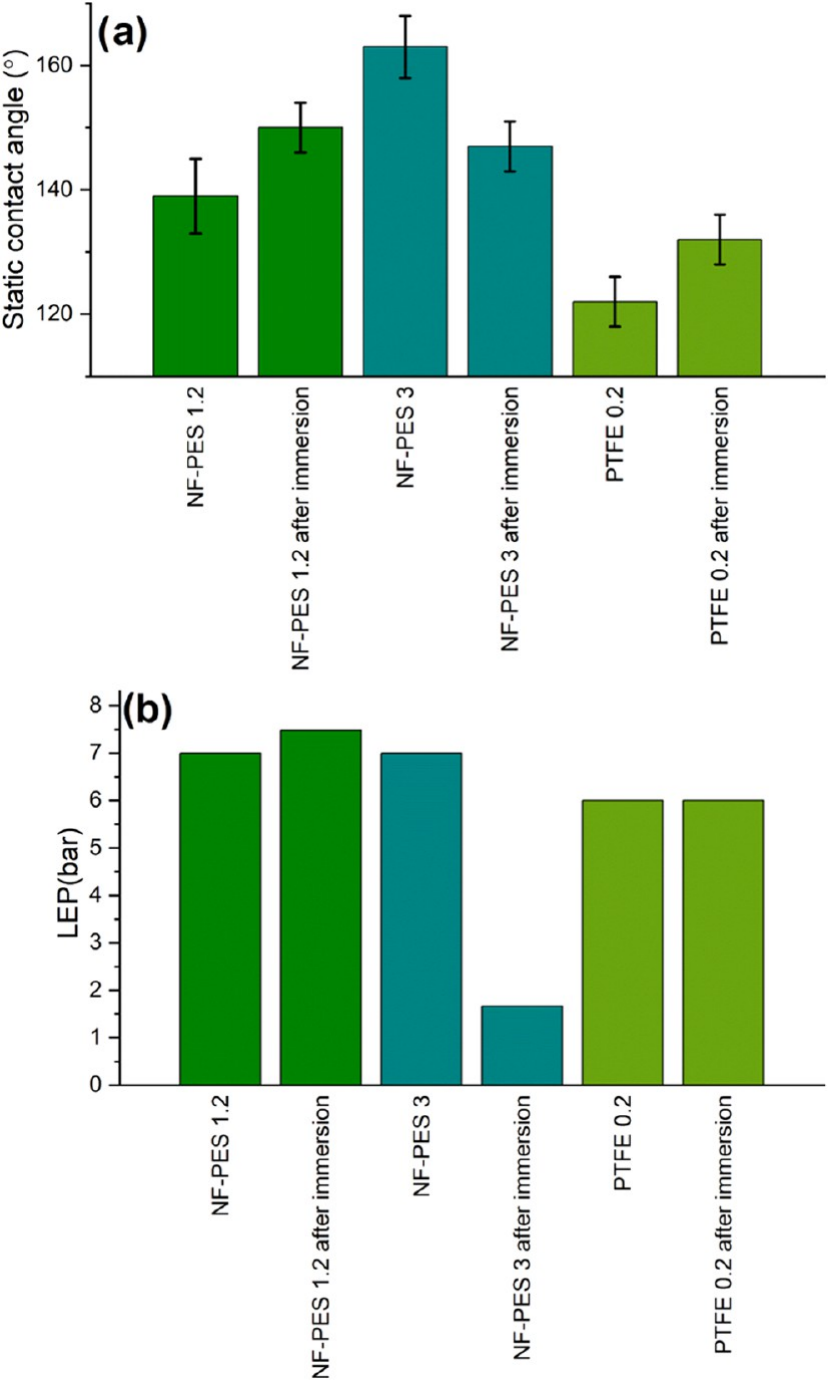
(a) Static contact angle of NF-PES and PTFE before and after the immersion test in HCl solution. (b) LEP value of NF-PES and PTFE before and after immersion in HCl solution.

To evaluate the effect of acid, NF-PES and PTFE membrane samples were immersed for 24 h in HCl pH 1 (Figure 5). Results obtained for NF-PES were compared with those for PTFE, which is a commercial membrane frequently used in membrane distillation.

**Figure 6:**
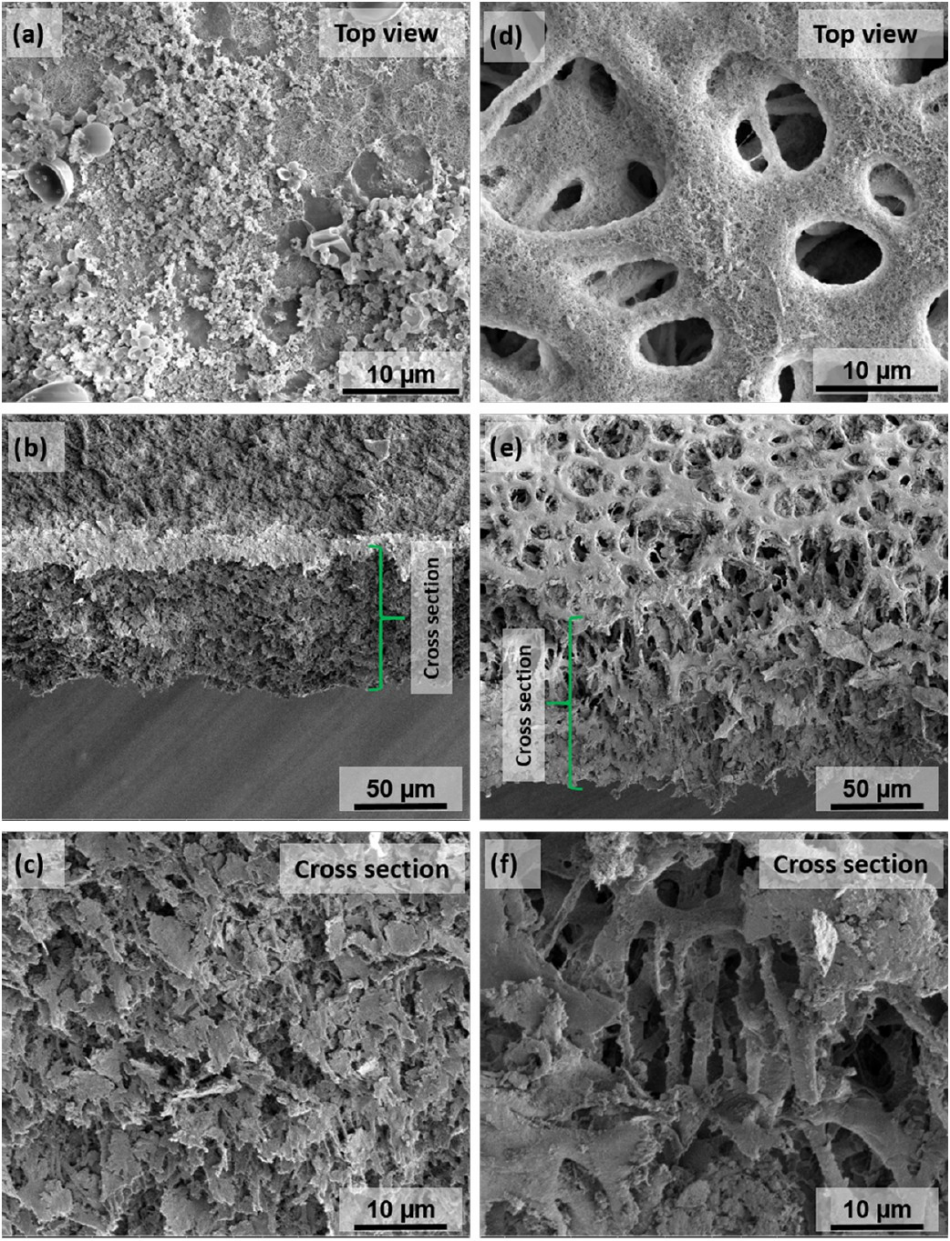
Scanning electron microscopy images of NF-PES membranes after immersion tests in HCl pH 1 for 24 h. (a–**c)** correspond to NF-PES 1.2. (d–f) correspond to NF-PES 3.

Minor variations were observed in the value of the static contact angle for all of the samples before and after the immersion tests in acid solution. With respect to the liquid entry pressure, NF-PES 1.2 membranes were not affected by pH 1. They showed an even higher liquid entry pressure than did the PTFE membranes. In contrast, the LEP value of NF-PES 3 decreased significantly after the immersion tests. This is in accordance with the increase of the pore size observed in the SEM image of the immersed sample. Therefore, the decrease in LEP can be just explained in terms of the change of the pore size of the core membrane upon exposure to acid. Statistical analysis of SEM images of the core PES 3 membrane showed that the pore size increases from (4 ± 2) to (8 ± 4) μm after the immersion tests in HCl. Not only the average value increases but also a wider distribution of pore sizes was found (Figure S2). The situation is different for NF-PES 1.2. It is tempting to say that the sample resists the acid solution, but by observing the morphology of the membranes by SEM imaging, it can be seen that the porous structure collapsed (Figure 6). In addition, cross-sectional SEM images show that the porous structure of NF-PES 1.2 membranes gets highly damaged by the acid treatment, while NF-PES 3 is also affected but not enough to completely destroy the porous structure. Therefore, measuring the wetting properties alone is not enough to state that the membranes keep their properties after exposure to the acid. It is not clear yet the mechanism through which the coating and core membrane are affected by the acid. Static contact angle measurements of NF-PES membranes exposed to HCl (pH 1) solution for 1 h already reveal some changes in the wetting properties, indicating that the membranes suffer modifications even after short times in contact with the acid liquid. In addition, Figure S3a shows an SEM image of an NF-PES 1.2 (after the immersion tests in acid for 24 h) recorded on a spot of the surface where it is visible how silicone nanofilaments turned into particles and a PES pore of the underneath membrane is being deformed. This may give hints about how the degradation process occurs. One may speculate that the acid medium hydrolyzes the chemical bond between the coating and the surface, which somehow induces a change in the morphology of the coating from nanofilaments into particles. Then, the underneath PES membrane gets exposed to the liquid, which causes damage to the porous structure of the PES membrane when it is in contact with the acid medium. Figure S3b shows an SEM image of an NF-PES membrane exposed to HCl at 80 °C, where even cracks appear as a result of the hostile treatment. This indicates that the membranes will not resist membrane distillation operation at high temperature with acid feeds. Finally, PTFE membranes did not show significant differences in the LEP value before and after immersion in acid medium. The results show that the coated membranes do not resist highly acidic conditions, and therefore, they may fail during membrane distillation operation with acid feeds.

**Figure 7:**
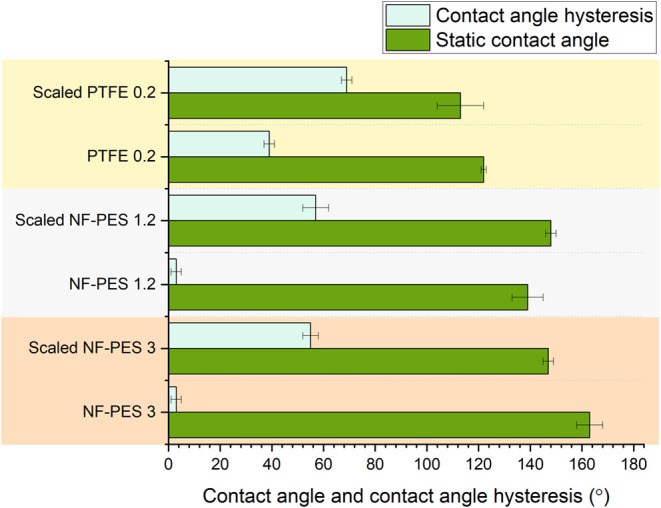
Wetting properties of NF-PES and PTFE membranes before and after immersion tests in synthetic seawater.

Immersion tests for 24 h in synthetic seawater (see composition in the Materials and Methods section) were conducted to study the resistance against scaling (Figure 7). All membranes showed an increase in the contact angle hysteresis and a decrease in the static contact angle after the immersion test, except NF-PES 1.2 that shows a slightly higher static contact angle after the immersion tests. Both, NF-PES 1.2 and 3 have the same static contact angle after the immersion tests, which is more than 30 degrees higher than for scaled PTFE standard membrane, while the contact angle hysteresis was between 12 and 14 degrees lower for NF-PES membranes than PTFE.

**Figure 8:**
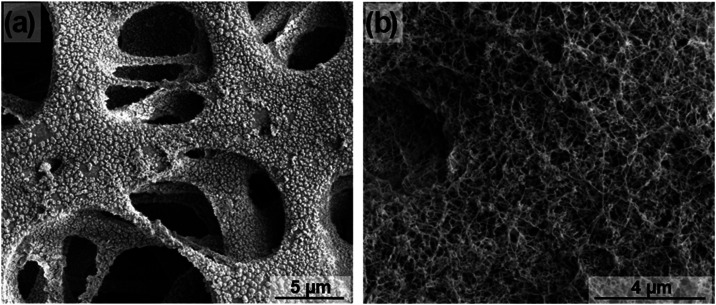
Scanning electron microscopy images of membranes after immersion test in synthetic seawater. (a) Original PES 3 and **(b)** nanofilament-coated PES 3.

In Figure 8, a scanning electron microscopy image of the original PES 3 membrane and the coated one after the immersion tests are shown. In the pristine PES membrane, carbonate particles crystallize probably due to the flat and hydrophilic nature of the polymeric surface. In contrast, no particles are observed on the coated membrane after the immersion tests. A possible mechanism to explain the higher resistance of coated membranes toward scaling is that the nanofilament coating provides a highly rough superhydrophobic surface where a majority of the solid–liquid contact is replaced by an air–water interface due to the air plastron forming. Therefore, there is less solid surface available for salt crystallization.

Encouraged by the fact that no particles were observed on the coated membranes and that they still showed better water repellency than PTFE after the immersion tests; air gap membrane distillation (AGMD) experiments were performed using synthetic seawater as feed solution.

**Figure 9:**
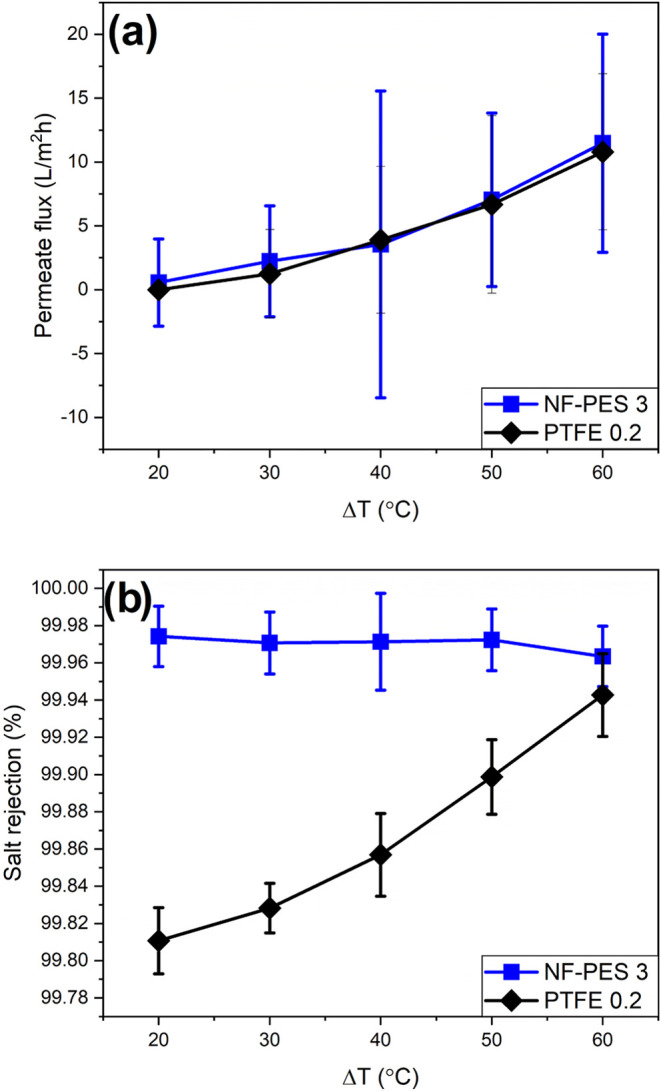
Air gap membrane distillation experiments varying the temperature difference between the feed and the coolant using synthetic seawater as the feed solution. (a) Permeation flux and (b) salt rejection percentage.

Figure 9 shows the permeate flux and salt rejection percentage as a function of the temperature difference between the coolant and the feed. The vapor permeation flux increased by increasing the temperature in the feed, as expected. Both NF-PES 3 and PTFE 0.2 display an average permeation flux of 11 L/m^2^h at the highest Δ*T* value. In a previous work published by our group, it was reported that NF-PES membranes exhibit larger permeation flux than PTFE and other commercial alternatives when using NaCl solutions as feed.^38^ Indeed, when conducting membrane distillation experiments in pilot scale modules using A4-sized NF-PES membranes, the permeation flux was up to 50% better than that in PTFE 0.2 for NaCl solutions (κ = 240 mS/cm).

When synthetic seawater was used as feed instead of NaCl solutions, the measured permeation flux for NF-PES membranes was similar to that for PTFE (Figure 9a). However, a better quality of the distillate was observed in the case of NF-PES membranes (Figure 9b). For NF-PES samples, the salt rejection percentage was constant over the whole range of Δ*T*. In contrast, the salt rejection decreased at low Δ*T* for PTFE membranes. In fact, partial wetting of the PTFE membrane was observed by eye inspection when removed from the membrane distillation setup. The decrease in the quality of the distillate for PTFE membranes when decreasing Δ*T* may be caused by the higher solubility of carbonates at low temperatures. In the case of partial wetting, more carbonate is expected to get in contact with the distillate when decreasing temperature.

**Figure 10:**
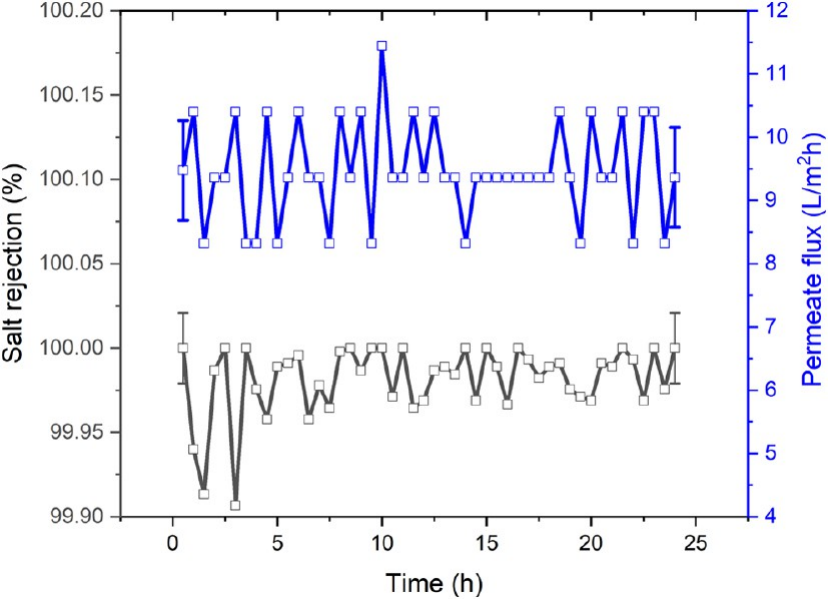
Air gap membrane distillation experiments for 24 h using NF-PES 3 at Δ*T* = 60 °C and synthetic seawater as feed solution.

Air gap membrane distillation experiments were conducted to study the performance of the nanofilament-coated PES membranes for 24 h using synthetic seawater as feed at Δ*T* = 60 °C (Figure 10). Both permeation flux and salt rejection were constant over the total operation time.

In principle, scale formation cannot be ruled out, since the immersion tests revealed changes in the wetting properties of the membranes. However, some outstanding features of the nanofilament-coated membranes should be pointed out: (i) after the immersion tests, NF-PES membranes still show better wetting properties than PTFE membranes exposed to the same conditions; (ii) no evidence of scaling substances crystallizing on NF-PES was found by scanning electron microscopy; (iii) constant salt rejection at different temperatures was obtained for NF-PES while a decrease in salt rejection at lower temperatures was found in PTFE membranes; and (iv) constant permeation flux and salt rejection were measured over time for NF-PES, which suggests no significant scaling effect during distillation operation.

### Biofouling Resistance

**Figure 11:**
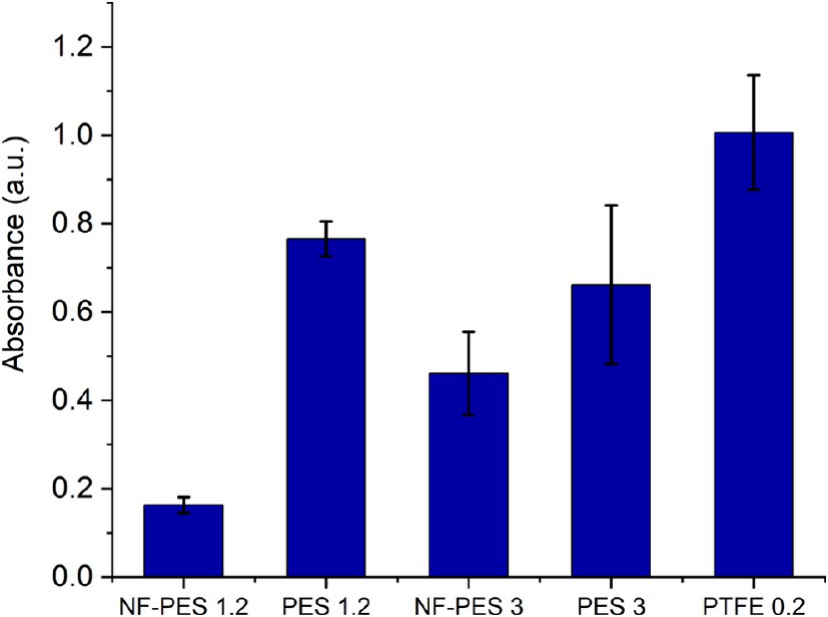
Absorbance at 600 nm of violet crystal in samples incubated with *Pseudomonas protegens*. The absorbance at the same wavelength in samples incubated in culture medium without bacteria was subtracted from the baseline.

Figure 11 shows the results of the absorbance of crystal violet for all of the different samples. Absorbance is a measure of amount of biofilm formed in the incubation well. The highest absorbance and thus most biofilm growth were measured for PTFE membranes. The absorbance of CV on PTFE membranes is followed by noncoated PES 1.2 and 3. For NF-PES 3 membranes, the biofilm growth was less than 50% of the growth on PTFE, while on NF-PES 1.2, the growth was less than 20% of the growth on PTFE. As mentioned in the previous discussion about scaling resistance, the application of the superhydrophobic coating minimizes the effective solid–liquid contact area by replacing a large part of the solid with an air plastron. This may introduce an advantage in comparison to standard membranes in terms of resistance to fouling processes.

**Figure 12:**
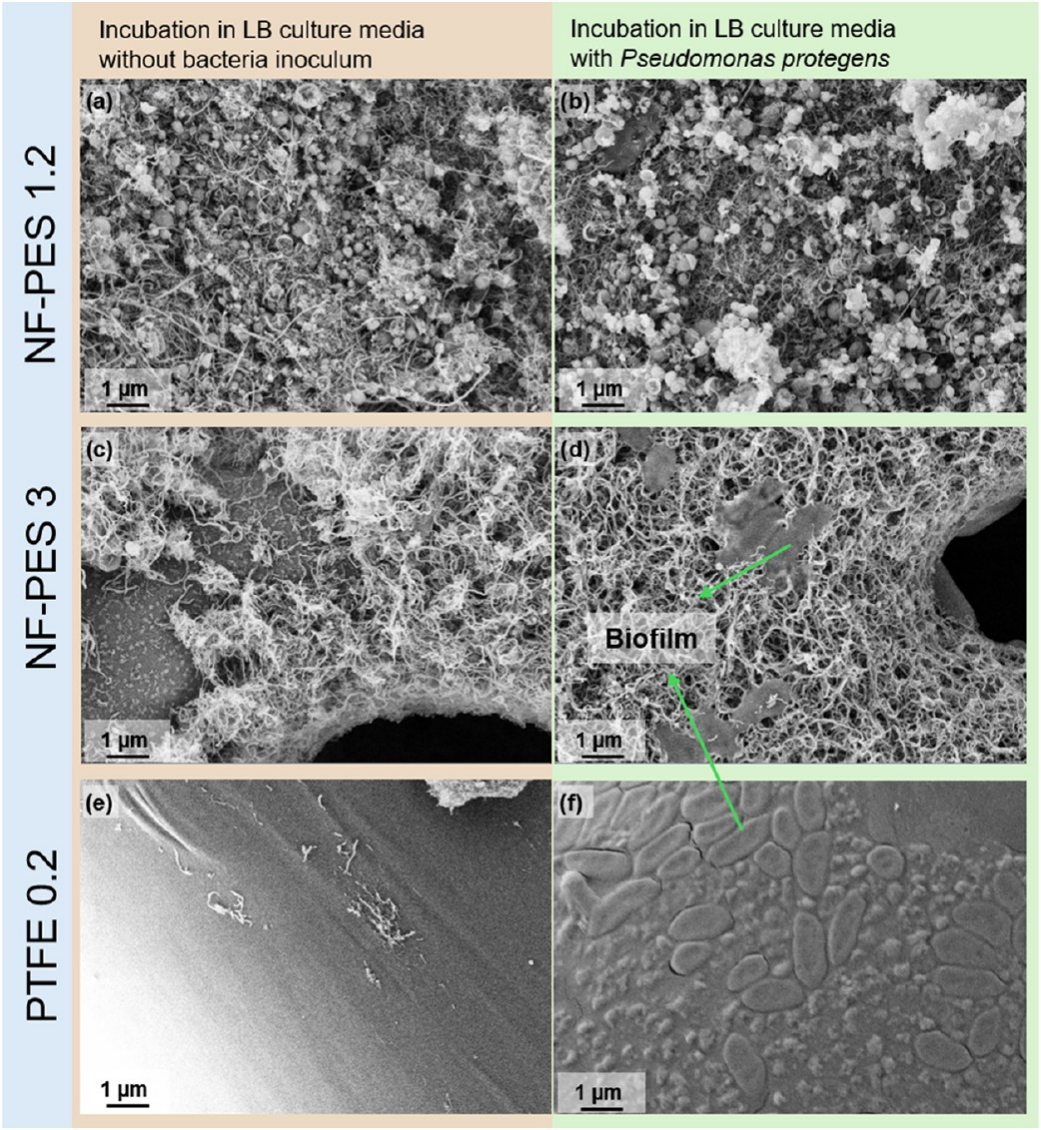
Scanning electron microscopy images of sterilized membrane samples incubated in pure culture medium (left) and sterilized samples incubated with bacteria in culture medium (right). (a, b) NF-PES 1.2, (c, d) NF-PES 3, and (e, f) PTFE 0.2.

**Figure 13:**
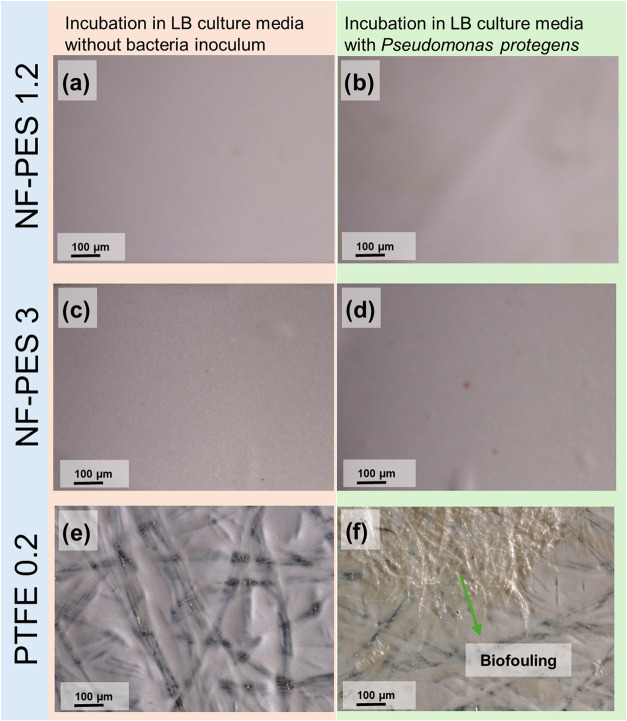
Optical microscopy images of sterilized membranes incubated in culture medium (left) and culture medium with bacteria (right). (a, b) NF-PES 1.2, (c, d) NF-PES 3, and (e, f) PTFE 0.2.

The absorbance measurements are confirmed by scanning electron micrographs (Figure 12) and optical microscopy images (Figure 13) of the membranes incubated in culture medium with and without bacteria. The combination of the UV sterilization procedure and incubation in a culture medium seems to affect the structure of the coating to some extent (small defects in coating for PES 3). The main outcome is that bacteria biofilm formation can be clearly seen on PTFE membranes, whereas just some cells were found on NF-PES 3 (most likely on damaged spots) and no biofilm was observed on NF-PES 1.2.

In summary, the commercial PTFE membranes are more susceptible to biofilm growth than the nanofilament-coated PES membranes. This provides a good proof of concept of the superior resistance of the NF-PES membranes against biofouling in comparison to that of PTFE. This is an interesting starting point for future work focused on evaluating biofouling during membrane distillation experiments.

### Membrane Distillation Performance with A Real Wastewater Sample

The wastewater sample we used as feed is a municipal aqueous digestate that contains between 800 and 1200 ppm of ammonia that can be recovered for its further utilization as fertilizer and for proper disposal of the remaining liquid. It also contains other contaminants, especially organic waste, which may cause wetting of the membrane due to organic fouling. We previously reported that nanofilament-coated membranes are resistant to organic fouling, and therefore, they may be suitable for ammonia recovery from the waste liquid.

Ammonia is more volatile than water. Therefore, even at low temperature differences between the feed and the coolant, ammonia is expected to be removed from the feed. The wastewater is a turbid brownish liquid, and after the distillation process, a transparent liquid is obtained as distillate. The presence of ammonia in the distillate could be verified by adding copper salt to it. Copper complexation by ammonia turns the distillate into a blue color (Figure S4).

**Figure 14:**
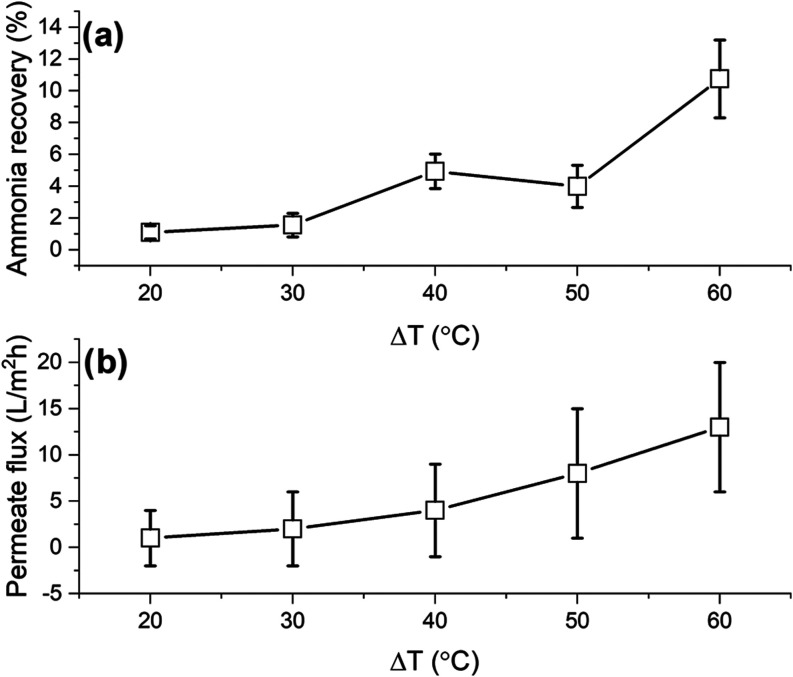
AGMD experiments as a function of Δ*T* using municipal wastewater containing ammonia as feed. (a) Ammonia recovery percentage. (b) Permeate flux.

Figure 14 shows the permeate flux and ammonia recovery percentage as a function of Δ*T* in AGMD. Permeate flux and ammonia recovery increase with an increase in the driving force. When comparing the permeate flux obtained with wastewater with the permeate flux obtained with synthetic seawater, the values are in the same order of magnitude at each Δ*T* value. The synthetic seawater solution has a conductivity value of 45 mS/m, whereas the real wastewater sample has a conductivity of 500 mS/m. This means that the performance of the membranes, in terms of permeation flux, is not affected when increasing the conductivity by 1 order of magnitude.

## Conclusions

Silicone nanofilament-coated membranes for membrane distillation could be prepared from an *n*-heptane solution. Heptane can replace the more toxic toluene as the solvent, which is a prerequisite for scaling up the coating reaction. The wetting properties of the resulting membranes showed that the material fulfills the basic requisites for being applied in membrane distillation. Acid, scaling, and biofouling resistance tests were conducted. The samples did not resist highly acidic conditions, mostly due to the low acid resistance of the core PES membrane. Scaling on coated membranes was not observed by SEM. The wetting properties of NF-PES, after immersion tests in scaling solutions, were better than those of PTFE standard samples. Distillation flux was in the same order of magnitude for NF-PES and PTFE, while the salt rejection was slightly lower for PTFE than NF-PES. No flux decline or decrease in salt rejection was observed after 24 h of operation using water with scaling substances. Biofouling resistance tests showed that biofilm growth was from 20% to 50% lower on NF-PES than PTFE. Finally, ammonia recovery from a real wastewater sample was accomplished, which constitutes a proof of concept of possible applications of the nanofilament-coated membranes.

We conclude that NF-PES membranes are suitable alternatives to fluorinated polymers for membrane distillation. Moreover, they exhibit better wetting repellency and resistance to different fouling sources in comparison to commercial PTFE membranes. The coating procedure has the potential for upscaling to industrial production. We are currently working on the implementation of a roll-to-roll process for large-scale fabrication, but more challenges need to be addressed, such as the necessary coating time to achieve the optimal wetting properties, and the effect of ambient humidity on the coating properties, which are the focus of future work.

## Supporting Information Available

SEM images of nanofilament-coated cellulose acetate membranes comparing the resulting morphology with and without plasma treatment (Figure S1). Pore size distributions of PES 3 membranes before and after immersion tests in acid showing how the pore size increases after immersion tests (Figure S2). SEM images of NF-PES membranes after immersion tests in HCl solution for 24 h at room temperature and at 80 °C showing degradation in the membrane structure (Figure S3). Pictures of the municipal wastewater used for MD experiments and the obtained distillate (Figure S4) (PDF)
