# Supplementary material for: Nanofilament-Coated Membranes with Enhanced Scaling and Biofouling Resistance for Membrane Distillation
Source: ACS Appl Mater Interfaces. 2025 Apr 15;17(16):24588–600. doi: 10.1021/acsami.5c01758 (PMC12022982; doi:10.1021/acsami.5c01758)
Supplement: Supplementary file 1 — am5c01758_si_001.pdf [file am5c01758_si_001.pdf]

Supporting Information

Nanofilament-coated membranes with enhanced scaling and biofouling resistance for membrane distillation

Mariana D. Sosa[1], Ivana K. Levy[2], Hans-Jürgen Butt[1], Michael Kappl[1]*

1. Department of Physics at Interfaces – Max Planck Institute for Polymer Research. Ackermannweg 10, Mainz, 55128 Germany.

2. Instituto de Química Física de Materiales, Ambiente y Energía (INQUIMAE). Consejo Nacional de Investigaciones Científicas y Técnicas (CONICET)-Universidad de Buenos Aires (UBA), Ciudad Universitaria, Pabellón 2, Ciudad Autónoma de Buenos Aires C1428EGA, Argentina.

*Corresponding author: kappl@mpip-mainz.mpg.de

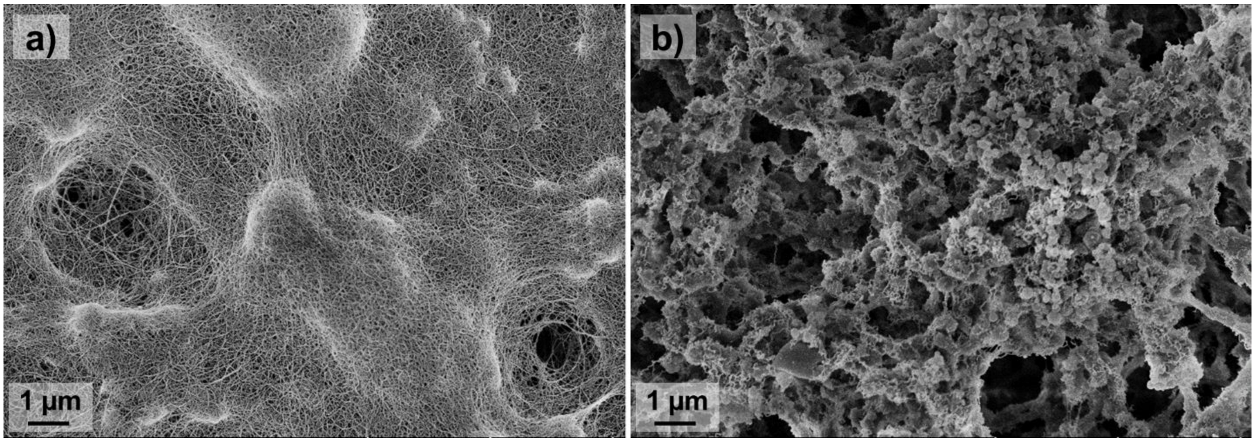

Figure S1. a) Nanofilament-coated cellulose acetate membrane. The coating procedure was performed without prior plasma activation showing that the intrinsic hydrophilicity of the core material and the presence of –OH active groups in the polymer structure are enough to ensure the growth of the filaments. **b)** Coated cellulose acetate membrane. In this case, plasma activation of the surface was done before the coating procedure. The hydrophilicity of the surface increases and no nanofilaments were observed in the coating layer.

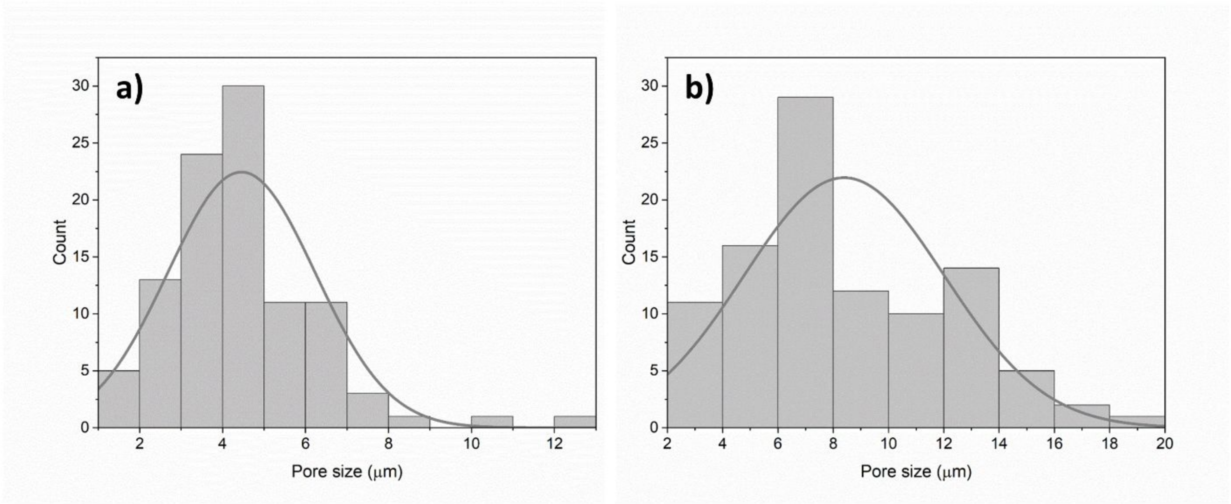

Figure S2. Pore size distribution of PES 3 membranes. **a)** Original PES 3, **b)** PES 3 after immersion test in HCl pH 1 for 24 hours. Both distributions were determined by analyzing SEM images of each membrane with ImageJ software. The histograms were plotted measuring the diameter of a total number of 100 pores for each sample.

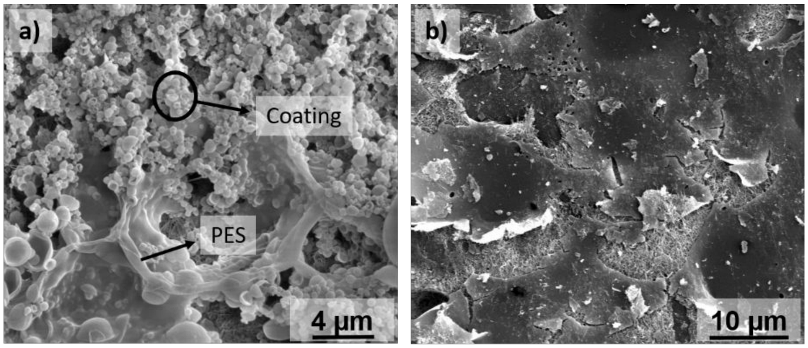

Figure S3. a) NF-PES after immersion test in HCl pH 1 for 24 hours. **b)** NF-PES after immersion test in HCl for 24 hours at 80ºC.

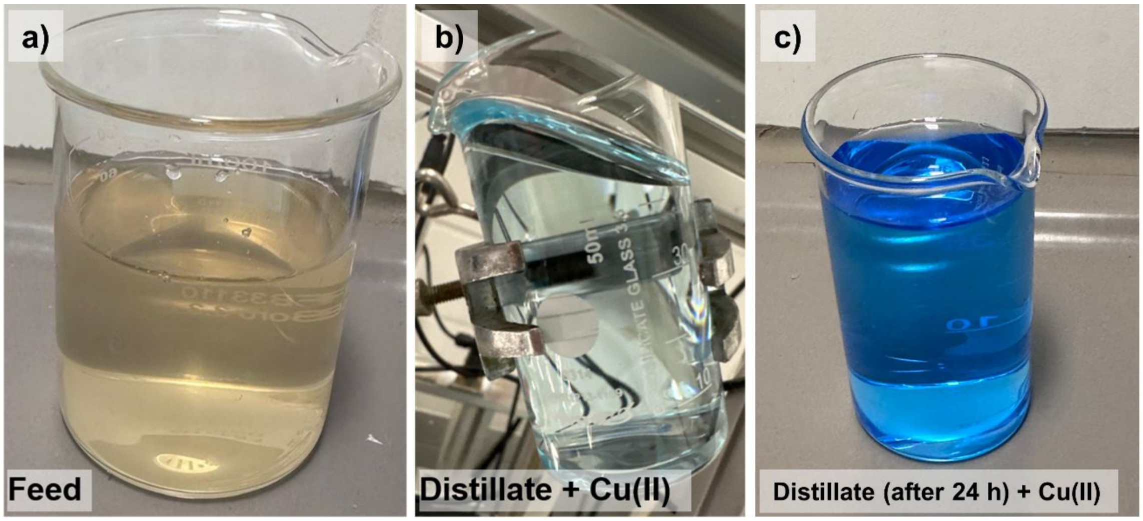

Figure S4. a) Municipal waste used as feed in air gap membrane distillation experiments. **b)** Distilled water obtained using nanofilament coated polyethersulfone membranes. A copper salt was added to evidence the presence of ammonia in the distillate. **c)** Distilled water containing ammonia after 24 hours of operation. A copper salt was added for generating the blue color that evidences the presence of ammonia in the distillate. The intensity of the blue color is correlated to ammonia concentration in the distillate.
